# Zinc toxicology following particulate inhalation

**DOI:** 10.4103/0019-5278.40809

**Published:** 2008-04

**Authors:** Ross G. Cooper

**Affiliations:** Division of Physiology, Birmingham City University, 704 Baker Building Franchise Street, Perry Barr, Birmingham B42 2SU, UK. E-mail: rgcooperuk@yahoo.com

**Keywords:** Breathing, exposure, fumes, occupation, respiration, zinc

## Abstract

The current mini-review describes the toxic effects of zinc inhalation principally in the workplace and associated complications with breathing and respiration. The International Classification of Functioning, Disability and Health Criteria were used to specifically select articles. Most of the commercial production of zinc involves the galvanizing of iron and the manufacture of brass. The recommended daily allowance for adults is 15 mg zinc/day. Metal fume fever associated with inhalation of fumes of ZnO is characterized by fatigue, chills, fever, myalgias, cough, dyspnea, leukocytosis, thirst, metallic taste and salivation. ZnCl_2_ inhalation results in edema in the alveolar surface and the protein therein the lavage fluid is elevated. Particular pathological changes associated with zinc intoxication include: pale mucous membranes; jaundice; numerous Heinz bodies; and marked anemia. Adequate ambient air monitors for permissible exposure limits, excellent ventilation and extraction systems, and approved respirators are all important in providing adequate protection.

## INTRODUCTION

Zinc is an essential mineral in cellular metabolism. It is a cofactor for the activity and folding of proteins.[1] Because of the pleiotropic effects of zinc on every aspect of cell physiology, zinc deficiency or excessive rise in its cellular concentration, can have catastrophic consequences and are linked to major patho-physiologies including diabetes and stroke.[1]

The plasma concentration of zinc is about 15 µmol/L, principally bound to albumin and a third of which is bound to α2-macroglobulin.[2] Zinc occupies 10-20 % of plasma, is a constituent of the human genome, acts as a site-specific antioxidant, acts as an active site in enzymes, and is essential for the action of insulin.[2]

Initial symptoms following exposure to concentrated hexite smoke, vomiting, cough and dyspnea, disappear after a few hours.[3] Thereafter, 48h later, acute respiratory distress syndrome appears requiring tracheal intubation and mechanical ventilation for eight days).[3] The patient left hospital 10 days after extubation. Spirometry at this time revealed a restrictive defect (vital capacity 50% predicted).[3]

Acute metal fume fever is commonly associated with zinc inhalation via welding, galvanizing, brass plating, dyes and electroplating.[4] Zinc severely impedes mitochondrial functions attenuating ATP production.[5] Potential health risks to workers exposed to zinc oxide (ZnO) and zinc chloride (ZnCl_2_) are significant.[6]

The aim of this mini-review was to describe the toxic effects of zinc inhalation principally in the workplace and associated complications with breathing and respiration.

## METHODS

The criteria used in the current mini-review for selecting articles to be included were both theoretically and practically motivated and adopted from the proposed criteria in The International Classification of Functioning, Disability and Health – ICF.[7] These criteria were as follows:

- Articles were chosen only with internationally recognized impact factors greater than 0.10
- Articles were chosen based upon the impact of lifestyle, stress and/or environmental factor/s predisposing zinc exposure
- Criteria for selection of literature used included yes-no responses to: the appropriateness of methodology; adequacy of subject numbers; specificity of sex and/or age of subjects; and statistically significant response rates to survey questionnaires
- The time frame used was principally 1990-2007 inclusive, although articles of extreme importance from earlier decades were used where appropriate
- A multi-factorial overview of the factors eschewed concerning zinc exposure was elucidated. It was presumed that collective articles detailing known factors of usage were not necessarily correlated with functionality and health.
- Compilation of materials for the mini-review started with published literature or easily accessible academic research
- The articles were accessible from on-line sources including PubMed and Medline.

## RESULTS

### Zinc inhalation and respiratory distress

The respiratory tract can be a significant port for heavy metal toxicity, inhalants often associated with lung cancer.[8] Most of the commercial production of zinc involves the galvanizing of iron and the manufacture of brass.[9] Others suggest exposure to zinc via fume inhalation during the active gas welding of steel coated with zinc protective layers.[10] One study demonstrated that exposure to fumes of silver solder containing cadmium was more likely to induce fever than fumes of zinc.[11] The recommended daily allowance for adults is 15 mg zinc/day. Ingestion of 1-2 g zinc sulphate (ZnS0_4_) produces emesis. Inhalation of high concentrations of ZnCl_2_ from smoke bombs detonated in close spaces may result in chemical pneumonitis and adult respiratory distress syndrome.[9] Metal fume fever linked with inhalation of fumes of ZnO is characterized by fatigue, chills, fever, myalgias, cough, dyspnea, leukocytosis, thirst, metallic taste and salivation. One study asked 12 healthy volunteers to inhale zinc oxide fumes through a mask for two hours at 0, 2.5 and 5 mg/m^3^ on separate days.[12] All but two developed a mild fever between 6 and 12 hours after exposure and all but one reported more symptoms (particularly fatigue, muscle ache and cough) after zinc oxide exposure at 5 mg/m^3^ than after exposure to the control furnace gas.[12] Another case study of a 25-year-old male welder with metal fume fever showed that he was suffering from aseptic meningitis with pericarditis, pleuritis and pneumonitis.[13] The condition is associated with invasion of neutrophils into the airways through stimulation of oxygen free radicals possibly associated with the associated pathogenesis.[14–16] In another study, ZnO welding fumes were associated with a marked dose-dependent increase in the number of polymorphonuclear leukocytes recovered in bronchoalveolar lavage fluid 22h following exposure, although it was not associated with a clinically significant change in pulmonary function or airway reactivity.[17] The authors suggest a cytokine-mediated mechanism. Haeme oxygenase gene expression occurs following inhalation of ZnO in welding fumes at concentrations equal to and below the current recommended threshold limit value of 5 mg/m^3^ ZnO.[18]

ZnO is a common constituent of particulate air pollution and if inhaled in fine or ultra-fine fractions at a concentration exceeding 500 µg/m^3^ for 2h can induce acute systemic effects.[19] There may be rapid production and secretion of metalloproteinases in the lung following heavy metal particle deposition including zinc.[20] Concurrent exposure to zinc and copper will result in significantly greater epithelial toxicity and stress.[21] Reduced glutathione levels have been associated with zinc-induced cytotoxicity.[22] Inhalation of ZnO at concentrations equal to and below a recommended threshold value of 5 mg/m^3^ results in the induction of target haeme oxygenase gene expression.[18] Significant changes in lavage fluid parameters have been described in guinea pigs and rats exposed to 2.5 mg/m^3^ ZnO.[23,24] Exposure of guinea pigs at three hr/day for five consecutive days to ultrafine ZnO at 7 mg/m^3^ resulted in a gradual decrease in total lung capacity and vital capacity.[25] Vital capacity, functional residual capacity, alveolar volume and diffusing capacity for carbon monoxide decrease following exposure to ZnO, although increases in flow resistance and decreases in compliance and total lung capacity return to normal by 72h.[26] Total lung capacity, vital capacity, functional residual volume, alveolar volume and diffusing capacity for carbon monoxide decrease following exposure to ZnO-SO_2_ mixtures and did not return to normal by 72h after the last exposure.[27] Further exposure resulted in greater decrements in functional residual capacity and residual volume.

ZnCl_2_ inhalation results in oedema in rats and measurements of alveolar surface protein in lavage fluid is variable, dose-dependent and maximal at three days, although at sub-lethal doses it regresses after seven days.[28]

### Zinc toxicity

Zinc compounds can produce irritation and corrosion of the gut, acute renal tubular necrosis and interstitial nephritis.[9] Inhalation would presumably contribute partially to pathological effects on the kidneys. Zinc toxicity can be treated with calcium disodium ethylenediaminetetraacetate, a chelator.[9] Zinc administration is able to attenuate some neurochemical, morphological and behavioral effects induced by pesticides like malathion.[29] Particular pathological changes associated with zinc intoxication include: pale mucous membranes; jaundice; the presence of numerous Heinz bodies; and marked anemia.[30]

### Influence on enzyme activity

Zinc-induced activation of epidermal growth factor receptor in human airway epithelial cells involves a loss of tyrosine phosphatase activities, potentially disrupting dephosphorylation reactions in cellular protein metabolism.[31] Cardiovascular blood coagulation impairments are likely following pulmonary zinc exposure and associated pulmonary injury and inflammation.[32] Concentrated ambient particle inhalation leads to tissue-specific increases in the activities of antioxidant enzymes including superoxide dismutase and catalase, suggesting that episodes of increased particulate air pollution trigger oxidant injurious effects and adaptive responses.[33] Zinc-induced toxicity has been linked to glutathione metabolism and cellular reduced glutathione activity.[34] Inhibition of glutathione reductase activity and the associated increase of oxidized glutathione may be responsible for zinc-mediated toxic cellular effects.[35] Reduction of glutathione is reduced in zinc-exposed cells and protection of glutathione (reduced) (GSH) oxidation by antioxidants and enhancement of cellular GSH content are mechanisms for diminishing the toxic cellular effects after exposure to zinc.[36] Exposure to 25-250 µmol ZnCl_2_ for 2h diminished protein synthesis and the decrease in total cellular glutathione is accompanied by an increased ratio of oxidized: reduced glutathione that was more pronounced in cells with low glutathione content.[37] Suppression of RNA and protein synthesis is due to the direct effect of zinc on these pathways.[38]

## DISCUSSION

Zinc forms a significant heavy metal occupational toxin, especially, within the metal processing industry. The lungs act as a trap for dust and therefore facilitate the diffusion of zinc into the blood stream. Workers need adequate protection from the vapors produced during metal working. It is in the interests of companies to provide very efficient protective measures in order to avoid litigation. In order to determine the Short-Term Exposure Limit Evaluation, a 15 min analysis of air within the workplace with a minimum of three measurements, the highest concentration recorded being taken as the estimate of the worker's exposure. Adequate ambient air monitors for permissible exposure limits, excellent ventilation and extraction systems, and approved respirators are all important in providing adequate protection. Although there is nothing published on the long-term exposure to ZnO fumes and the EU does not regard zinc dust as a human carcinogen, it does not negate the fact that adequate precautions need to be taken to prevent exposure.
